# Supplementary material for: The complete assembly of human LAT1-4F2hc complex provides insights into its regulation, function and localisation
Source: Nat Commun. 2024 May 2;15:3711. doi: 10.1038/s41467-024-47948-4 (PMC11065870; doi:10.1038/s41467-024-47948-4)
Supplement: Supplementary file 1 — Supplementary Information [file 41467_2024_47948_MOESM1_ESM.pdf]

Supplementary Information

The complete assembly of human LAT1-4F2hc complex provides insights into its regulation, function and localisation

Di Wu [1, 2, *], Renhong Yan [3], Siyuan Song [1, 2], Andrew K. Swansiger [4], Yaning Li [5], James S. Prell [4], Qiang Zhou [6] and Carol V. Robinson [1, 2, *]

1. Department of Chemistry, University of Oxford, Oxford, OX1 3QZ, UK.
2. Kavli Institute for Nanoscience Discovery, University of Oxford, Oxford, OX1 3QU, UK.
3. Department of Biochemistry, Key University Laboratory of Metabolism and Health of Guangdong, School of Medicine, Southern University of Science and Technology Shenzhen 518055, Guangdong Province, China.
4. Department of Chemistry and Biochemistry, University of Oregon, Eugene, Oregon 97403-1253, United States.
5. Beijing Advanced Innovation Center for Structural Biology, Tsinghua-Peking Joint Center for Life Sciences, Tsinghua University, Beijing, 100084, China.
6. Research Center for Industries of the Future, Zhejiang Key Laboratory of Structural Biology, School of Life Sciences, Westlake University; Institute of Biology, Westlake Institute for Advanced Study; Westlake Laboratory of Life Sciences and Biomedicine, Hangzhou 310024, Zhejiang Province, China.

*Correspondence: di.wu2@chem.ox.ac.uk; carol.robinson@chem.ox.ac.uk

This PDF file includes:

Supplementary Figure 1 to 18

Supplementary Methods

Supplementary References

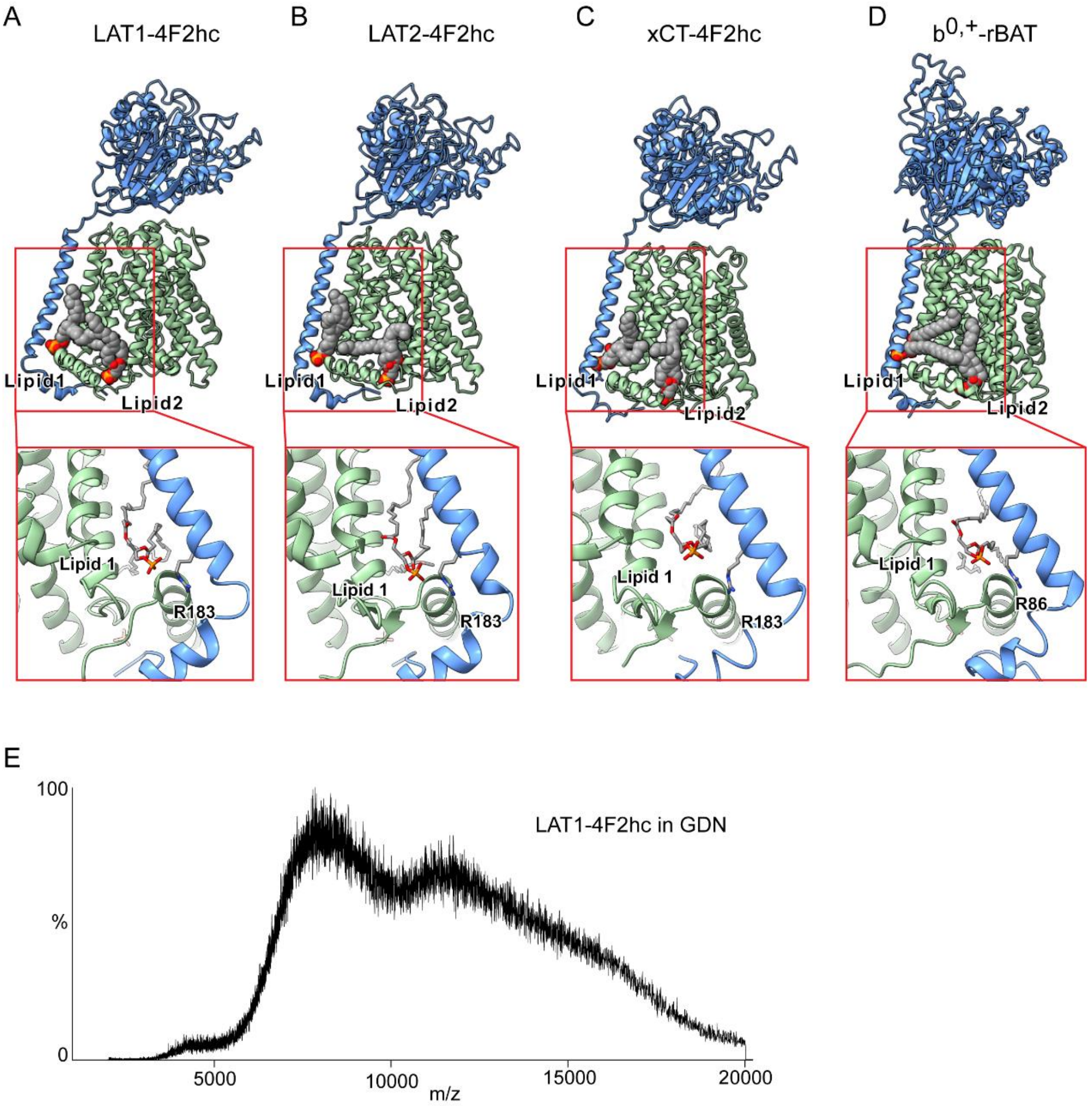

Supplementary Figure 1. Structural comparison of LAT1-4F2hc (PDB:6IRT), LAT2-4F2hc (PDB:7CMI), xCT-4F2hc (PDB:7EPZ) and $b^{0,+}$-rBAT (PDB:6LI9) complexes with two phospholipids binding to the interface of the heterodimer in each case. The head group of phospholipid 1 (lower boxes) interacts with 4F2hc-R183 (panel A, B and C blue, grey lines) and rBAT-R86 (panel D blue, grey lines). E) Native mass spectrum of LAT1-4F2hc in glyco-diosgenin (GDN) micelles reveals a large unresolved peak without definition of charge states.

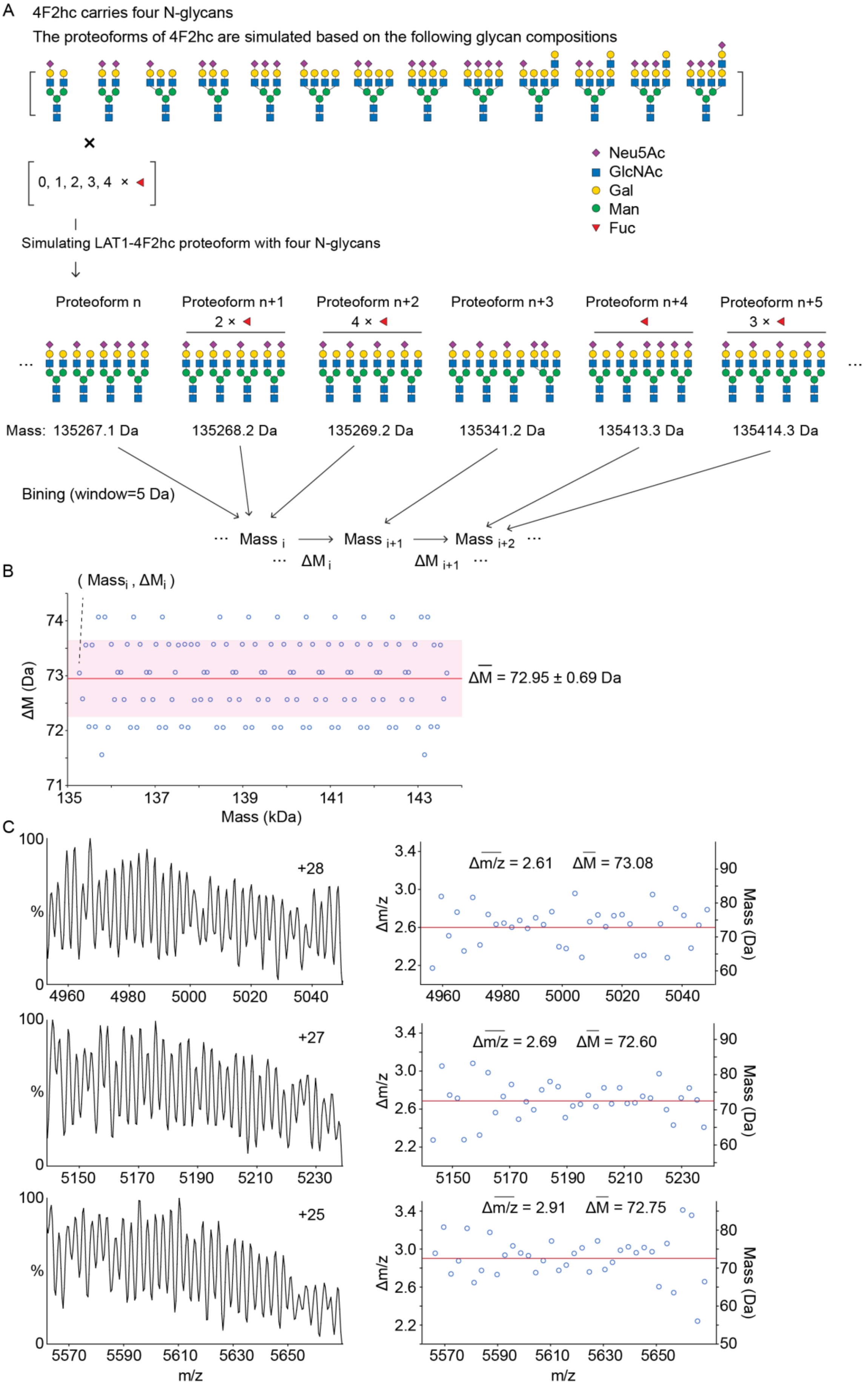
A
4F2hc carries four N-glycans
The proteoforms of 4F2hc are simulated based on the following glycan compositions
×
0, 1, 2, 3, 4 ×
Neu5Ac
GlcNAc
Gal
Man
Fuc
Simulating LAT1-4F2hc proteoform with four N-glycans
Proteoform n
Proteoform n+1
2 ×
Proteoform n+2
4 ×
Proteoform n+3
Proteoform n+4
Proteoform n+5
3 ×
Mass: 135267.1 Da
135268.2 Da
135269.2 Da
135341.2 Da
135413.3 Da
135414.3 Da
Bining (window=5 Da)
Mass i
Mass i+1
Mass i+2
ΔM i
ΔM i+1
B
(Mass_i , ΔM_i)
ΔM (Da)
ΔM = 72.95 ± 0.69 Da
Mass (kDa)
C
+28
Δm/z = 2.61
ΔM = 73.08
+27
Δm/z = 2.69
ΔM = 72.60
+25
Δm/z = 2.91
ΔM = 72.75
%
Δm/z
Mass (Da)
m/z

Supplementary Figure 2. Probing the glycoforms of LAT1-4F2hc. A) Simulation of LAT1-4F2hc proteoforms. The N-glycan structures with different numbers of Fuc, Neu5Ac and Gal-GlcNAc units were extracted from a previous bottom-up MS analysis of LAT1-4F2hc [10], and used for simulation of LAT1-4F2hc proteoforms with four N-glycans at the intact protein level. The molecular weight of each proteoform is calculated based on the mass of protein backbone and combination of the four N-glycans. The simulated proteoforms are firstly sorted in ascending order, and then binned with a window of 5 Da. The mass difference between each proteoform (ΔM) is calculated and plotted as a scatter graph in panel B. The average mass difference between each proteoform is calculated as 72.95 ± 0.69 Da. C) The charge states of +28, +27, and +25 in the spectra of heterodimeric LAT1-4F2hc. The Δm/z between the adjacent peaks is calculated and plotted (scatter plots right-hand side). The results show that each peak differs by ~73 Da for each charge state. Based on these simulations this assessment suggests that these peaks are resolved glycoforms with different monosaccharide compositions.

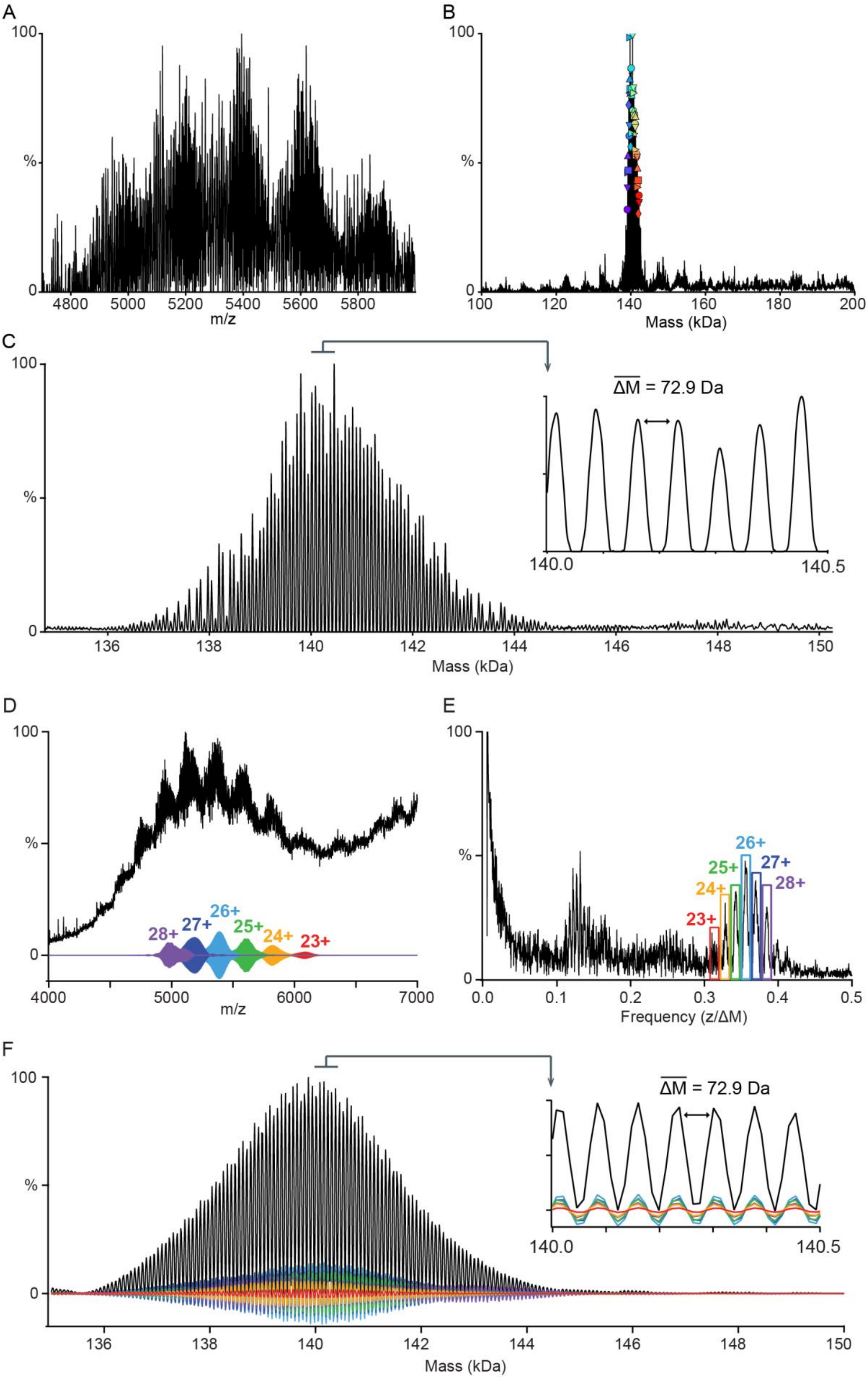

A
B
C
D
E
F
m/z
Mass (kDa)
Frequency (z/ΔM)
$\overline{\Delta M}$ = 72.9 Da
28+
27+
26+
25+
24+
23+

Supplementary Figure 3. Deconvolution of LAT1-4F3hc complex using UniDec (panel A, B and C) and iFAMs (panel D,E and F) software. A) The processed mass spectrum with background subtraction (line subtraction: 1). This spectrum is used for UniDec deconvolution. B) The zero-charged spectrum (deconvolved) of LAT1-4F2hc complex with Mass Distribution Smoothing function (mass differences: 72.95; mass smooth width: 10). The major peaks are ~140 kDa. The UniScore (average peaks score) is 56.35 with R^2 of 0.99989. C) The deconvolved spectrum showing LAT1-4F2hc peaks ranging from 136 to 150 kDa. The inset spectrum shows the average mass difference ($\overline{\Delta M}$) is 72.9 Da. D) The unprocessed native mass spectrum (black peaks) overlayed with the iFAMs-reconstructed LAT1-4F2hc peaks with charge +23 to +28 (colored peaks). E) The corresponding FT spectrum shows the charge-state-specific peaks (highlighted with colored boxes). F) The deconvolved zero-charged spectrum. The inset spectrum shows the average mass difference ($\overline{\Delta M}$) is 72.9 Da.

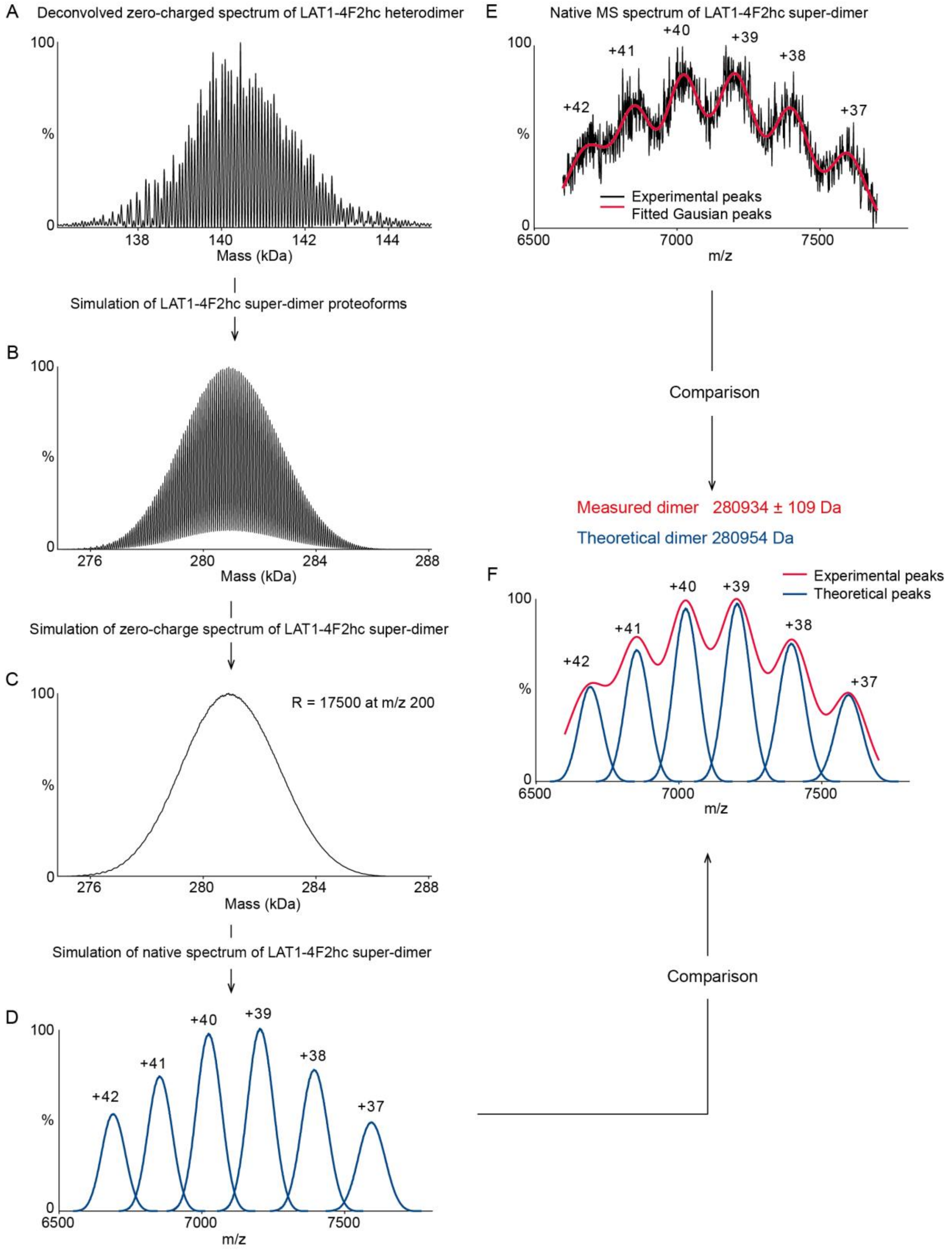

Supplementary Figure 4. Simulation of theoretical LAT1-4F2hc super-dimer based on the heterodimeric LAT1-4F2hc spectrum. (A) The masses and abundances of LAT1-4F2hc proteoforms were extracted from the UniDec-deconvolved zero-charged spectrum. (B) The LAT1-4F2hc super-dimer proteoforms were simulated using a binomial distribution model based on the LAT1-4F2hc heterodimer information and plotted as a zero-charged spectrum. (C) To compare the simulated and

experimental LAT1-4F2hc super-dimer spectra, the resolution (m/Δm) of the simulated LAT1-4F2hc super-dimer spectrum was reduced to 2900. This resolution represents the typical resolving power (R=17500 at m/z 200) of a Q-Exactive UHMR mass spectrometer for analyzing a 280 kD membrane protein. (D) The theoretical native mass spectrum of LAT1-4F2hc super-dimer was then simulated at R=17500, (E) The experimental spectrum was compared with the simulated spectrum. (F) There is no significant difference between the theoretical (280954 Da) and experimental molecular weights (280934±109 Da) of LAT1-4F2hc super-dimers. The higher baseline of the experimental mass spectrum of the LAT1-4F2hc super-dimer implies that the super-dimer may still carry multiple detergents/solvent ions.

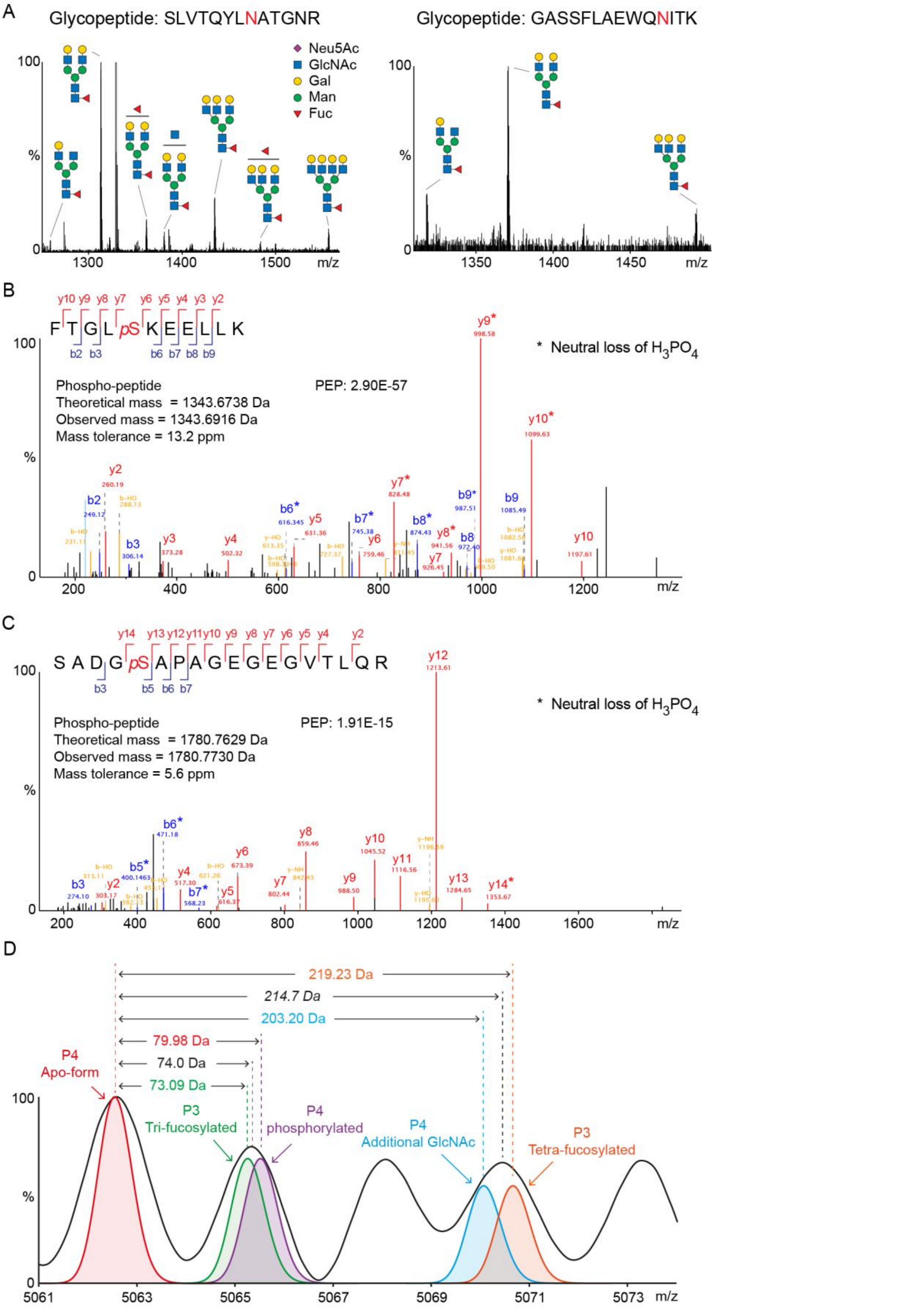

Supplementary Figure 5. Glycoproteomics and phosphoproteomics of the desialylated LAT1-4F2hc heterodimer. A) MS spectrum of glycopeptides encompassing 4F2hc-N365 (left panel) and 4F2hc-

N424 (right panel). The N-glycan compositions are labelled on the corresponding peaks. All N-glycans are core-fucosylated, in line with the previous glycomics and glycoproteomics analysis of the recombinant 4F2hc subunit [10]. B) MS/MS spectrum of a phosphopeptide encompassing 4F2hc-S165. C) MS/MS spectrum of a phosphopeptide containing LAT1-S35. The peaks with neutral loss of 98 Da (H_3PO_4) are labelled with asterisks. D) Native MS spectrum of the desialylated LAT1-4F2hc complex (5061-5073 m/z, charge state +27). The theoretical peaks of P4 proteoforms (apo, phosphorylated and with additional GlcNAc) and P3 proteoforms (tri- and tetra-fucosylated) were simulated and plotted. The simulation show that the tri- and tetra- fucosylated P3 proteoforms overlap with the P4 proteoforms with phosphorylation and an additional GlcNAc, respectively.

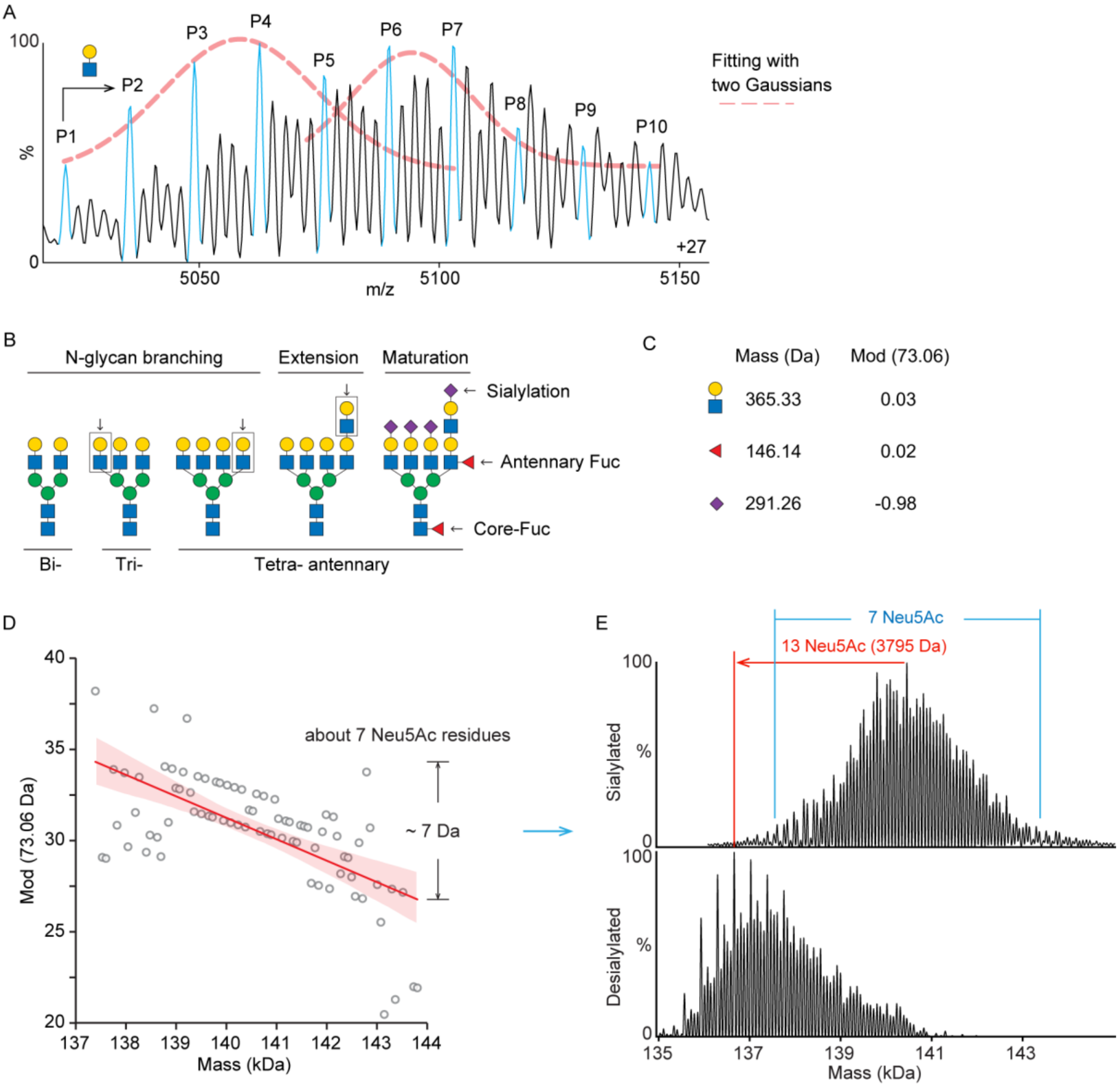

Supplementary Figure 6. Analysis of N-glycan branching and sialylation on LAT1-4F2hc. A) Annotation of the N-glycan branching events on the desialylated LAT1-4F2hc heterodimer (charge state +27). The major peaks differing by a $GlcNAc_1Gal_1$ unit are labelled with P1 to P10. Two Gaussian curves can be fitted to the P1 to P10 proteoforms, highlighted with dashed red lines. B) N-glycan branching ($GlcNA_1Gal_1$), fucosylation (Fuc_1) and sialylation ($Neu5Ac_1$) are the three main features of the N-glycan microheterogeneity in the LAT1-4F2hc complex. C) Calculating the modulus of N-glycan branching, fucosylation and sialylation. D) The correlation between the masses of sialylated LAT1-4F2hc and their modulus (mass mod 73.06). The trend line (linear regression) is shown (red line) with 95% confidence interval (red shaded area). From the modulus analysis, we found that the largest LAT1-4F2hc proteoform carries seven more Neu5Ac residues than the smallest proteoform. E) Comparison of sialylated and desialylated LAT1-4F2hc complexes. Neuraminidase treatment results in a loss of ~3795 Da (~13 Neu5Ac residues) from LAT1-4F2hc complex. These data suggest that the fully glycosylated LAT1-4F2hc complexes carry 9 to 16 Neu5Ac residues on its four N-linked glycans.

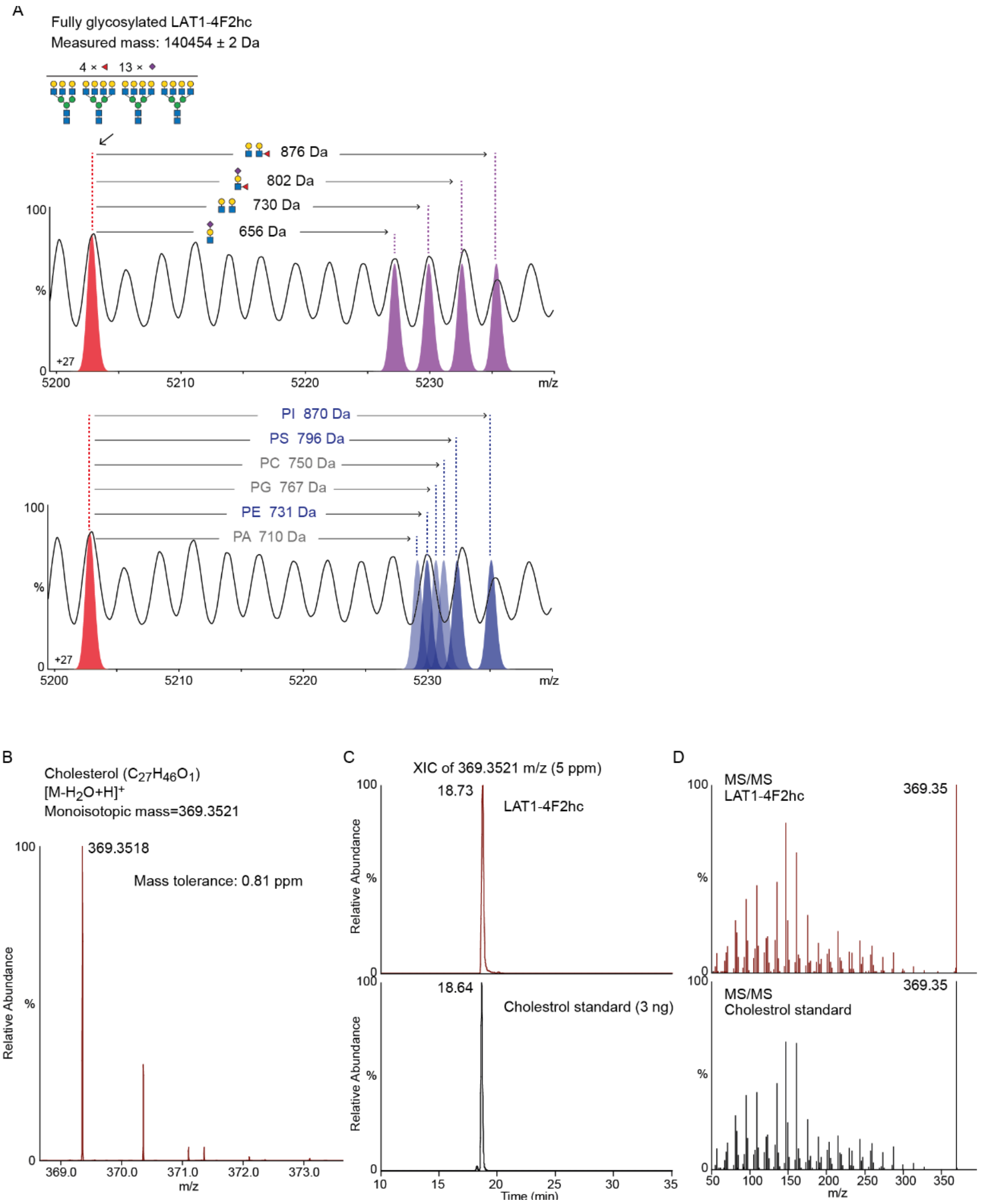

Supplementary Figure 7. A) Assignment of LAT1-4F2hc proteoforms and lipid adduct peaks. The isotopic envelopes of glycosylated LAT1-4F2hc, with different glycan structures and bound lipids, were simulated and plotted alongside the native mass spectrum of LAT1-4F2hc (charge state +27). Due to the inherent isotopic peaks of large protein complex and the limited resolution of the mass spectrum, LAT1-4F2hc heterodimer proteoforms and potential lipid-bound peaks overlap in the spectrum. B) Full mass spectrum of cholesterol molecule ($[M\text{-}H_2O+H]^+$, 369.3518 m/z) identified in LAT1-4F2hc sample. C) Comparison of the extracted ion chromatograms of 369.3521 m/z in LAT1-4F2hc sample and cholesterol standard (on-column amount of 3 ng). D) The comparison of tandem mass spectra of 369.35 m/z in LAT1-4F2hc sample and cholesterol standard. These suggest cholesterol as a co-purified lipid of LAT1-4F2hc assemblies.

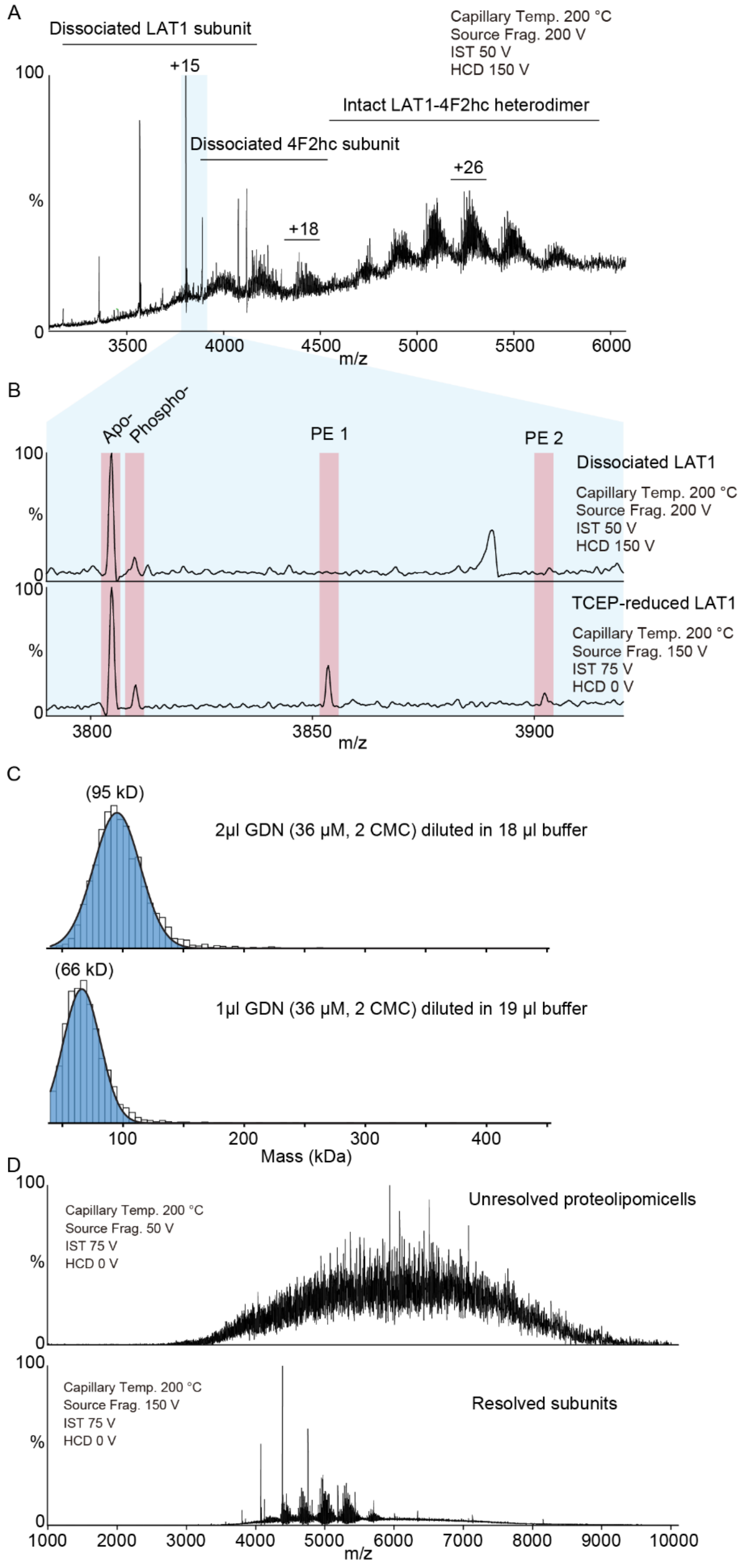

Supplementary Figure 8. A) Mass spectrum of gas-phase dissociated desialylated LAT1-4F2hc without disulphide bond reduction. The majority of the LAT1-4F2hc heterodimer remains intact after gas-phase collisional activation. The native MS parameters, including capillary temperature, source fragmentation

energy (Source Frag.), In-source trapping energy (IST) and HCD energy (HCD) are labelled. B) Expanded view of the native mass spectra of gas-phase dissociated LAT1 (top panel) and TCEP-reduced LAT1 (bottom panel). The higher collision energy used to dissociate LAT1 from the LAT1-4F2hc heterodimer also removes bound phospholipids. C) Mass photometry analysis of glyco-diosgenin (GDN) micelles. GDN micelles with different volumes as shown were diluted into phosphate-buffered saline (PBS) to give a total volume of 20 µl. In both measurements the GDN micelles are < 100 kDa in line with a previous report [11]. This result suggests that empty GDN micelles after dilution do not affect mass photometry analysis for large membrane protein complexes (> 100kDa). D) Native mass spectra of TCEP-reduced LAT1-4F2hc heterodimer under different activation energies. We observed unresolved proteolipomicells at lower activation energy (source fragmentation 50 V), and well-resolved LAT1 and 4F2hc subunits under higher activation energy (source fragmentation 150 V).

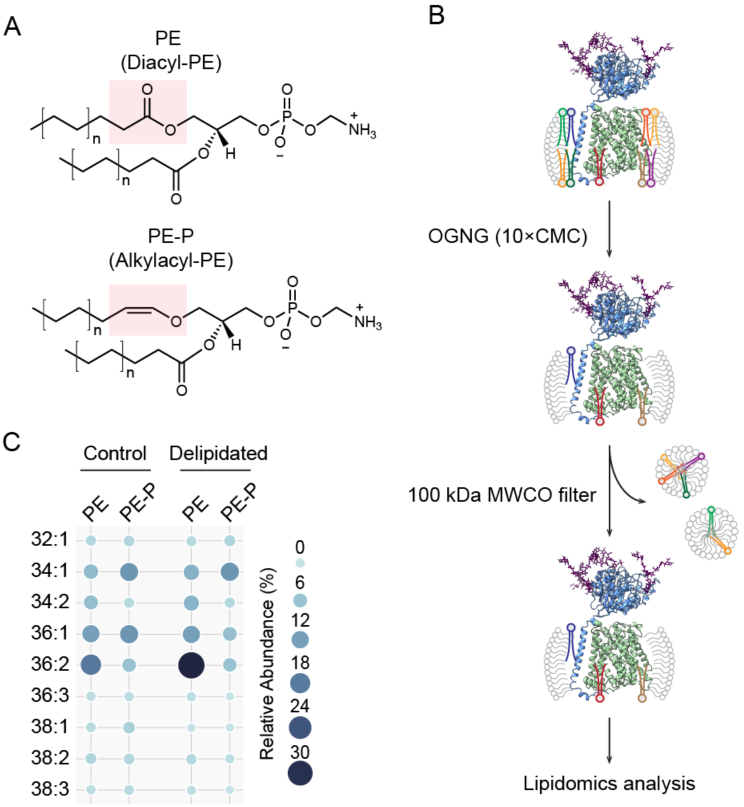

Supplementary Figure 9. A) Structures of the diacyl-PE and alkyacyl-PE (PE-P) lipids. B) Schematic workflow of in-solution delipidation. In brief, the LAT1-4F2hc assemblies were incubated with 10×CMC OGNG to release loosely bound lipids. The dissociated lipids were further removed after buffer-exchange using 100 kDa MWCO filters (Amicon, Millipore). The delipidated LAT1-4F2hc protein complexes were then analyzed with MS-based lipidomics. C) Relative abundances of PE and PE-P in LAT1-4F2hc samples before and after delipidation (control and delipidation respectively).

A

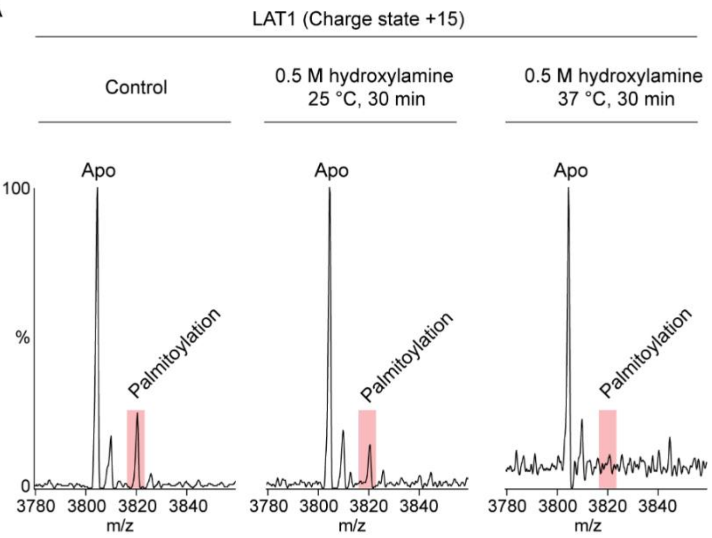

B

AA Position	Score	Cutoff
C187	0.7489	0.6785
C458	0.7051	0.6785

C

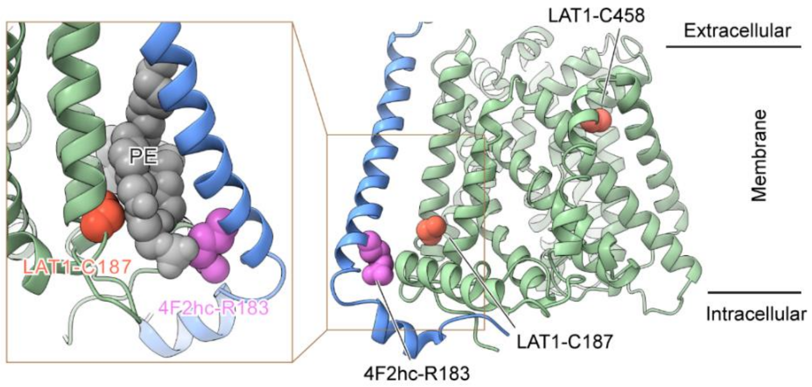

Supplementary Figure 10. A) Validation of LAT1 palmitoylation observed in mass spectra as an adduct (shaded pink) after expression of the LAT1-4F2hcR183 mutation. Hydroxylamine was used to break thioester bonds between the palmitoyl group and sulfhydryl group at pH 7.0 [12]. Following TCEP reduction mass spectra of LAT1 (charge state +15) were recorded as a function of temperature (25 °C or 37 °C). The palmitoylated proteoform of LAT1 was found to reduce after hydroxylamine treatment, more effecfively at the higher temperature. B) Prediction of palmitoylation site of LAT1 using GPS-Palm software [1]. Only two Cys residues in human LAT1 (C187 and C458) are predicted to carry S-palmitoylation. C) Structural illustration of LAT1-C187, LAT1-C58, 4F2hc-R183 and the interfacial PE lipid. LAT1-C187 is in close vicinity of 4F2hc-R183, whereas LAT1-C458 is distal to 4F2hc-183. The interfacial PE is between LAT1-C187 and 4F2hc-R183. Therefore, we propose that the mutation of LAT1-R183L, which abolishes interfacial PE binding, induces palmitoylation on LAT1-C187.

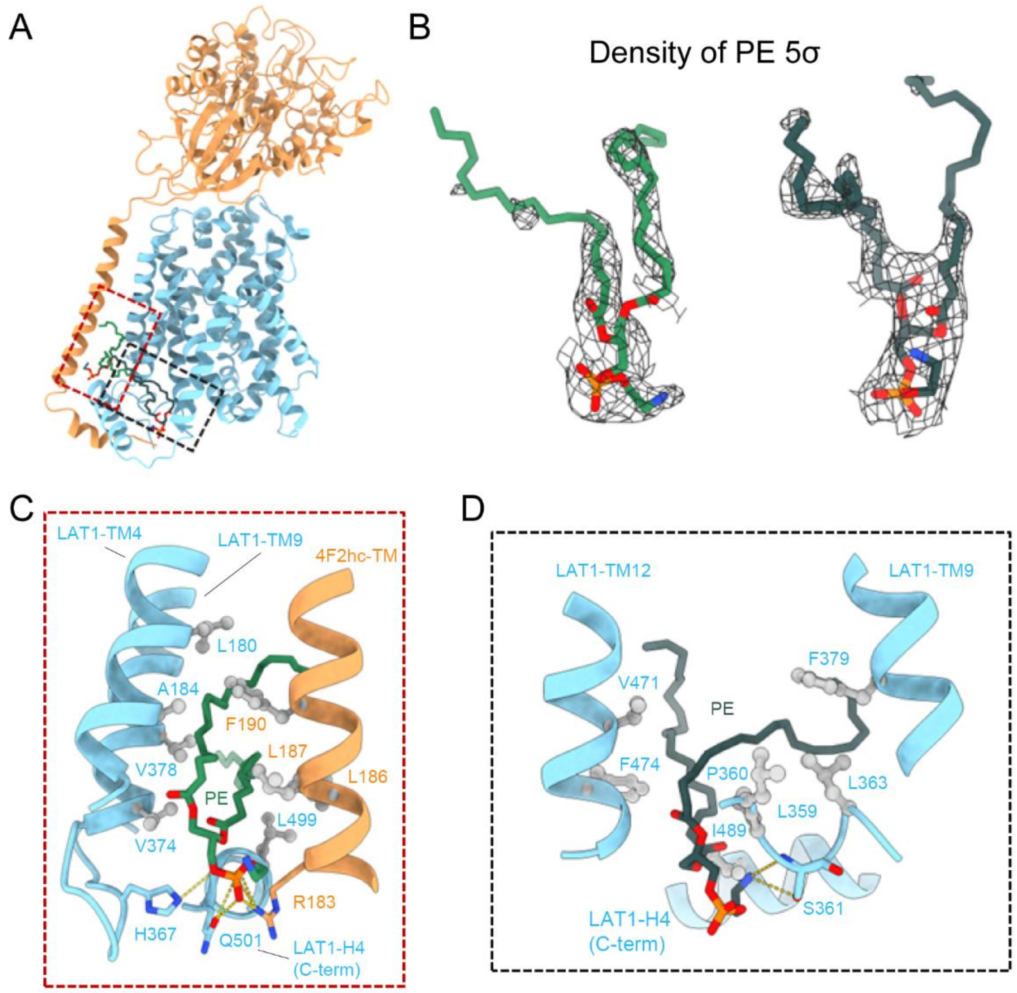

Supplementary Figure 11. A) Structure of the LAT1-4F2hc heterodimer determined by cryo-EM with two PE molecules refined in the density. B) Densities for two PE molecules, shown in gray mesh, are contoured at 5σ. C) Structural illustration of the interfacial PE molecule interacting with LAT1 (TM4, TM9 and H4) and 4F2hc (TM). D) Structural illustration of the PE in the hydrophobic pocket formed by LAT1-TM9, TM12 and H4.

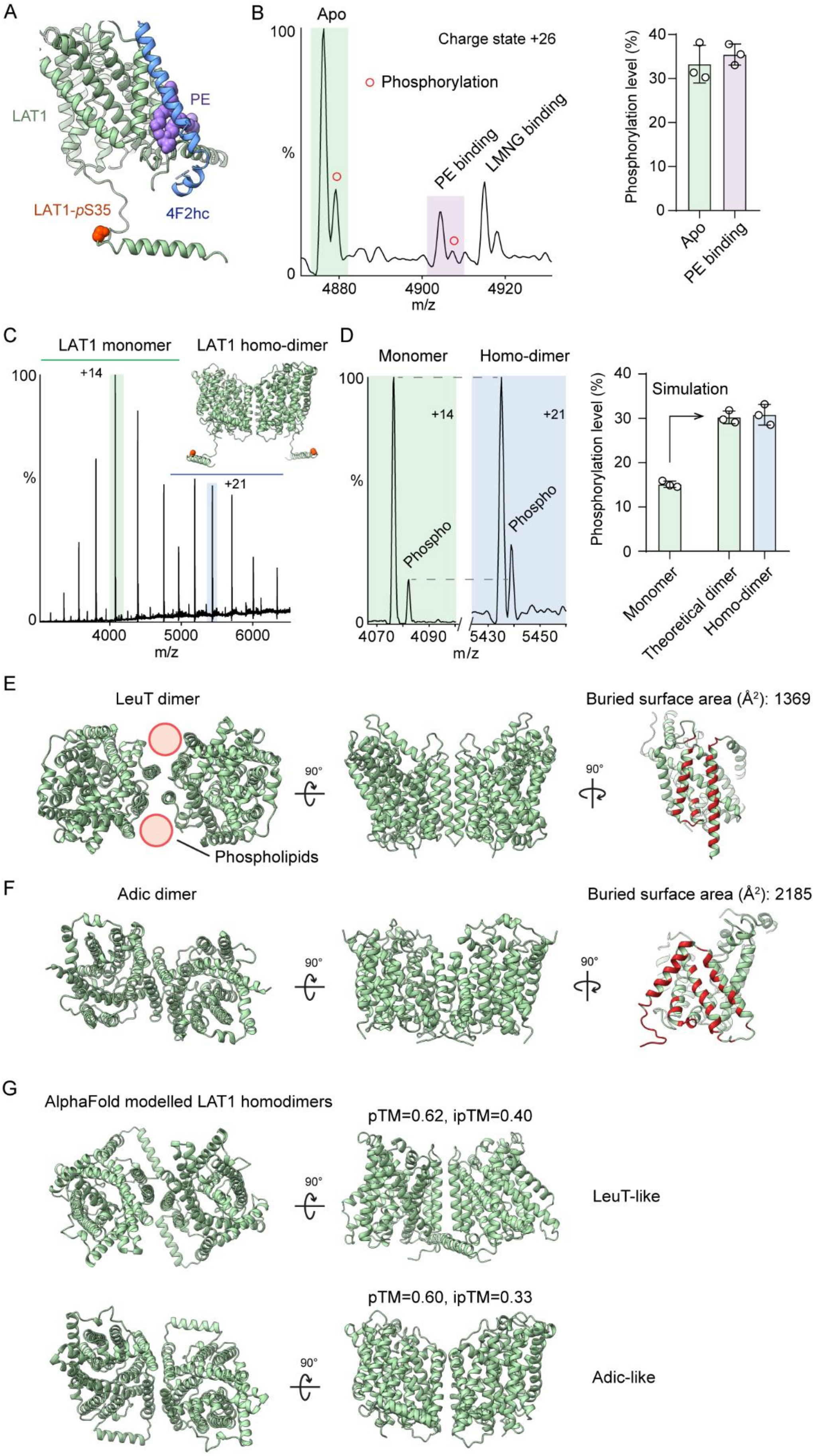

A
LAT1
PE
LAT1-pS35
4F2hc
B
Apo
Charge state +26
Phosphorylation
PE binding
LMNG binding
100
%
0
4880
4900
4920
m/z
Phosphorylation level (%)
40
30
20
10
0
Apo
PE binding
C
LAT1 monomer
LAT1 homo-dimer
+14
+21
100
%
0
4000
5000
6000
m/z
D
Monomer
Homo-dimer
+14
+21
Phospho
Phospho
100
%
0
4070
4090
5430
5450
m/z
Simulation
Phosphorylation level (%)
40
30
20
10
0
Monomer
Theoretical dimer
Homo-dimer
E
LeuT dimer
90°
90°
Buried surface area (Å²): 1369
Phospholipids
F
Adic dimer
90°
90°
Buried surface area (Å²): 2185
G
AlphaFold modelled LAT1 homodimers
pTM=0.62, ipTM=0.40
90°
LeuT-like
pTM=0.60, ipTM=0.33
90°
Adic-like

Supplementary Figure 12. Analysis of possible phospho-regulation of LAT1 dimerisation and interactions with PE. A) Structural illustration of LAT1-4F2hc complex with PE binding and phosphorylation on LAT1-S35. B) The spectrum of non-glycosylated LAT1-4F2hc 4M complex without and with PE binding (charge state +26). The peaks corresponding to phosphorylated proteoforms are labeled (red circles). The phosphorylation levels of apo- and PE-bound forms of LAT1-4F2hc 4M complex are plotted with error bars showing mean ± standard deviation from three independent experiments (dots). C) Native mass spectrum of LAT1 monomers and homo-dimers. D) Comparison of phosphorylation levels of monomeric (charge state +14) and homo-dimeric LAT1 (charge state +21). The theoretical phosphorylation level of the LAT1 homo-dimer was simulated based on the abundance of phosphorylated LAT1 monomer. Bars show mean ± standard deviation from three independent experiments (dots). E) Structure of the LeuT dimer. Interfacial phospholipid binding is critical for LeuT dimerization and the amino acids in the homo-dimer interface are highlighted in red. The buried surface area was calculated using PDBePISA server [13]. F) Structure of Adic dimer with interfacial amino acid residues involved in homodimerization highlighted in red. The buried surface area is higher for Adic than LeuT. G) AlphaFold-Multimer [4] predicted two models for the LAT1 homodimer with ipTM score greater than 0.3. The upper and lower models are LeuT-like and Adic-like LAT1 homodimers. The observation of lipid-free LAT1 homo-dimer supports the proposal that the LAT1 homodimerization is Adic-like with a greater buried surface area removing the requirement for lipid binding [14].

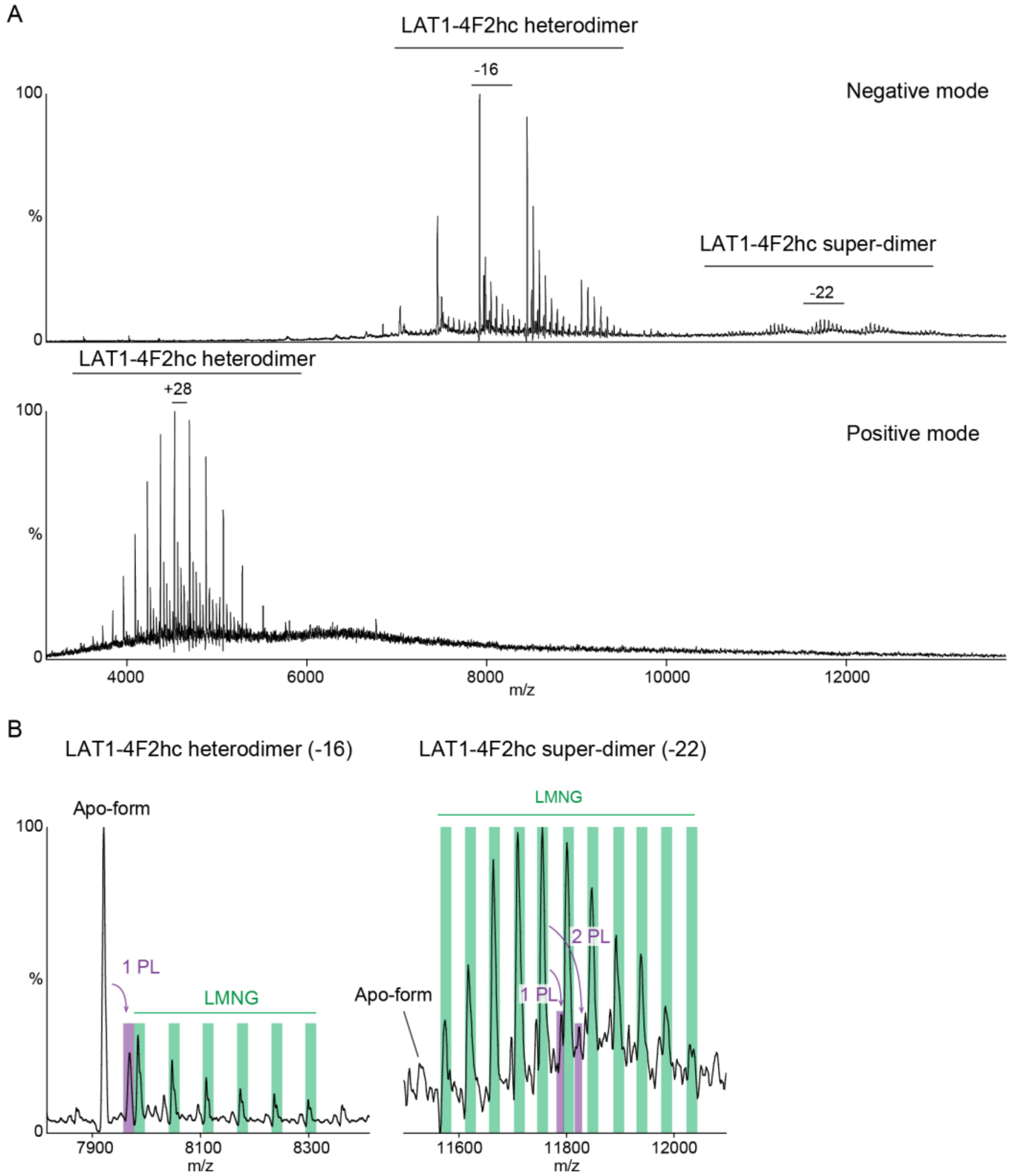

Supplementary Figure 13. Native MS analysis of the non-glycosylated 4M LAT1-4F2hc super-dimerization. A) Native mass spectra of non-glycosylated LAT1-4F2hc in the negative and positive ion modes (top and bottom panel). We only observed the non-glycosylated super-dimer in the negative mode likely due to the reduction in charge (+28 and -16 of the heterodimeric forms) and the effects of negative charge on stabilising the super dimer interface. B) Annotation of LAT1-4F2hc heterodimer and super-dimer peaks. The phospholipid (PL) and detergent binding peaks are highlighted in purple and green, respectively. The phospholipid adduct is ~ 730 Da, assigned to PE. Both one and two lipid binding peaks are observed in the absence of glycosylation.

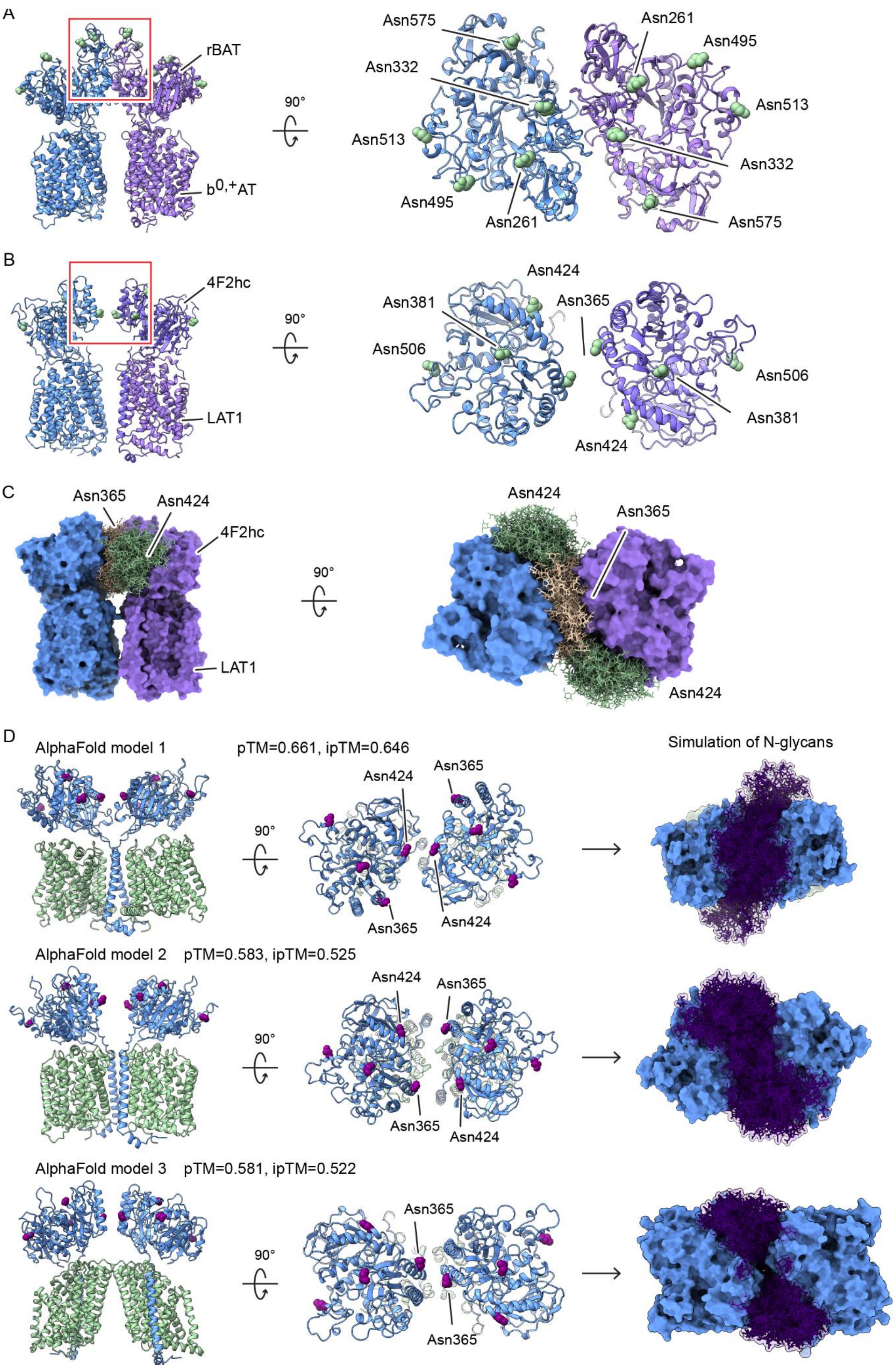
A
rBAT
b0,+AT
90°
Asn575
Asn332
Asn513
Asn495
Asn261
Asn261
Asn495
Asn513
Asn332
Asn575
B
4F2hc
LAT1
90°
Asn424
Asn381
Asn365
Asn506
Asn506
Asn381
Asn424
C
Asn365
Asn424
4F2hc
LAT1
90°
Asn424
Asn365
Asn424
D
AlphaFold model 1
pTM=0.661, ipTM=0.646
Simulation of N-glycans
90°
Asn424
Asn365
Asn365
Asn424
AlphaFold model 2 pTM=0.583, ipTM=0.525
90°
Asn424
Asn365
Asn365
Asn424
AlphaFold model 3 pTM=0.581, ipTM=0.522
90°
Asn365
Asn365

Supplementary Figure 14. A) Structure of $b^{0,+}$AT-rBAT super-dimer (PDB:6LI9). The glycosylated Asn residues are highlighted in green. The two rBAT subunits contact each other and facilitate the super-dimerization of $b^{0,+}$AT-rBAT complex. B) The homology-modelled structure of LAT1-4F2hc super-dimer using Pymol with PyMOD plugin [15]. The glycosylated Asn residues are highlighted in green. C) Illustration of the LAT1-4F2hc super-dimer homology model with possible N-glycan conformers on Asn365 and Asn424. The N-glycan conformers on Asn365 and Asn424 are highlighted in light brown and green, respectively. D) AlphaFold2-multimer models of LAT1-4F2hc super-dimer. Three super-dimer models were obtained with iPTM (interface predicted template modelling score) > 0.5. Glycosylated Asn365 and Asn424 are proximal to the super-dimer interface in all three predicted models. The AF-multimer predicted LAT1-4F2hc super-dimer models are similar to the homology-modelled structure (panel B).

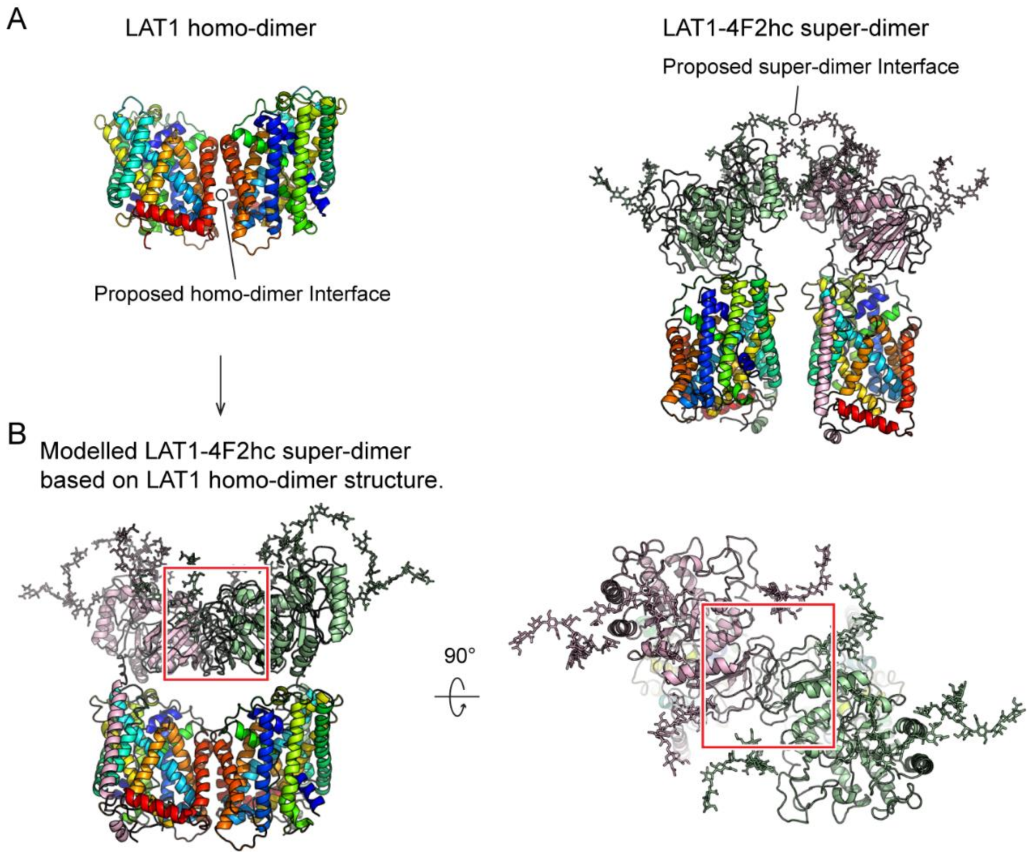

Supplementary Figure 15. Illustration of the different LAT1 interfaces in the LAT1 homodimer and the LAT1-4F2hc super-dimer models. The LAT1 homo-dimer was modelled using AlphaFold-Multimer. The LAT1-4F2hc super-dimer was proposed following homology-modelling. The attachment of 4F2hc disrupts the LAT1 homodimer due to steric clashes between the ECD of each 4F2hc subunit (highlighted, red box).

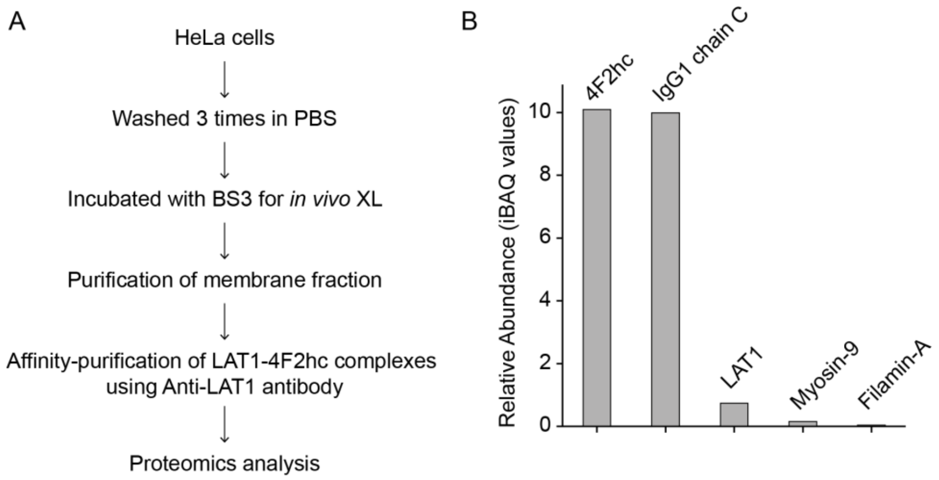

Supplementary Figure 16. Probing endogenous LAT1-4F2hc super-dimerization on cell membrane. A) Flowchart of affinity purification-MS of *in vivo* crosslinked LAT1-4F2hc assemblies. B) Top-five identified proteins based on their iBAQ (intensity-based absolute quantification) value using proteomics analysis. The large hydrophobic transmembrane domains of LAT1 could not be assessed by trypsin digestion followed by proteomics analysis. Therefore, the iBAQ value of LAT1 evaluated by proteomics is significantly lower than that of 4F2hc.

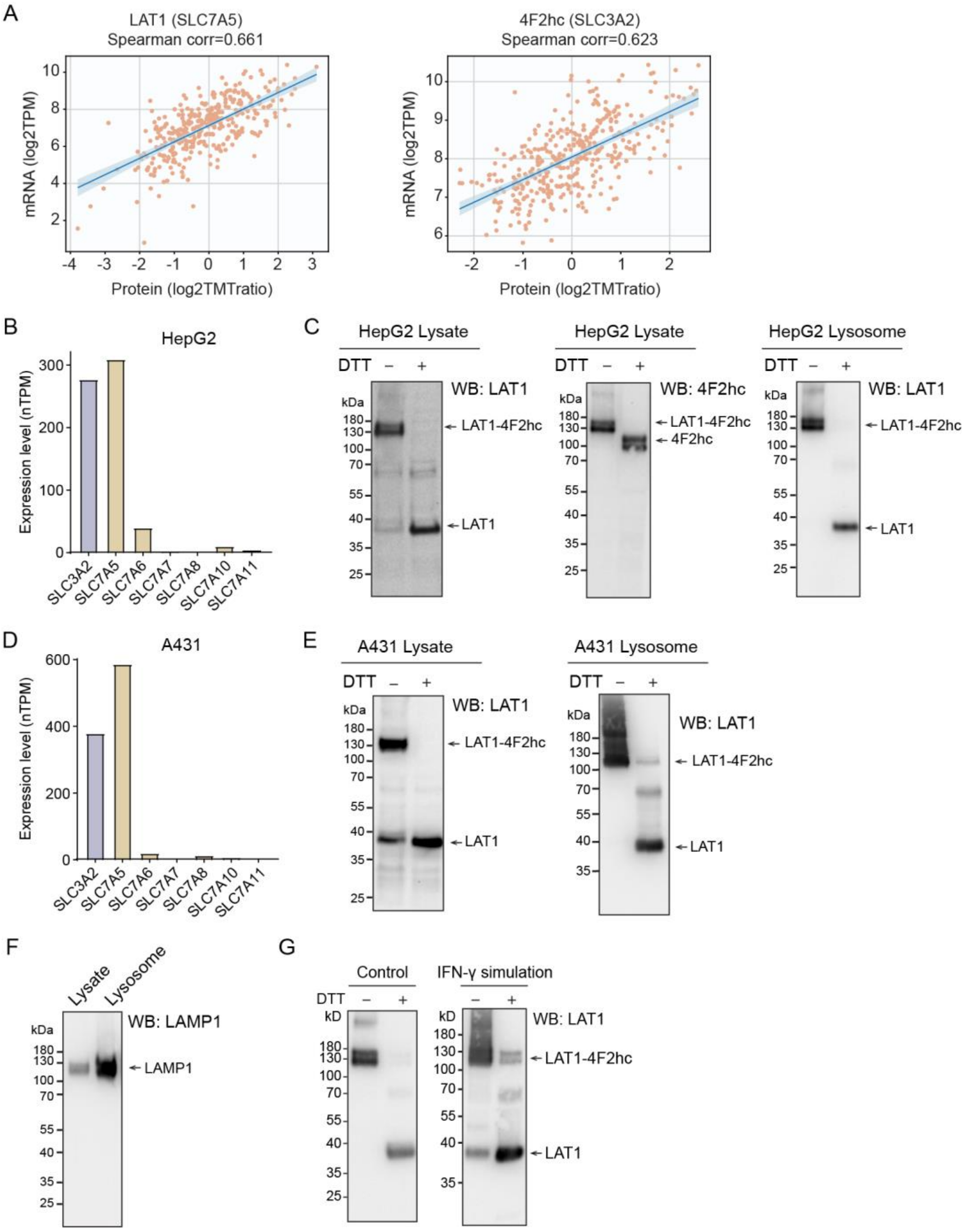

Supplementary Figure 17. A) Correlation of the mRNA and protein expression levels of LAT1 (SLC7A5) and 4F2hc (SLC3A2) in 375 cancer cell lines. The relative protein expression levels (measured in TMT (tandem mass tag) ratio) and mRNA levels (measured in normalized transcripts per million, nTPM) of LAT1 and 4F2hc were extracted from the Cancer Cell Line Encyclopedia [16] and plotted as a scatter plot. The linear-fit trendline is highlighted (dark blue). The shaded area indicates the 95% confidence interval. B) Expression level of 4F2hc (SLC3A2) and SLC7As in HepG2 cells. C) Western blotting analysis of the LAT1-4F2hc assembly in HepG2 cells. The experiment was repeated once. Source data are provided as a Source Data file. D) Expression level of 4F2hc (SLC3A2) and SLC7As in A431 cells. E) Western blotting analysis of the LAT1-4F2hc assembly in A431 cells with and without DTT. The experiment was repeated once. Source data are provided as a Source Data file.

F) Western blotting using LAMP1 antibody verifies that the lysosomal fraction was purified from HeLa cells. The experiment was repeated once. Source data are provided as a Source Data file. G) Western blotting of LAT1-4F2hc complexes in purified lysosomes from HeLa cells without and with IFN-γ stimulation. The experiment was repeated once. Source data are provided as a Source Data file.

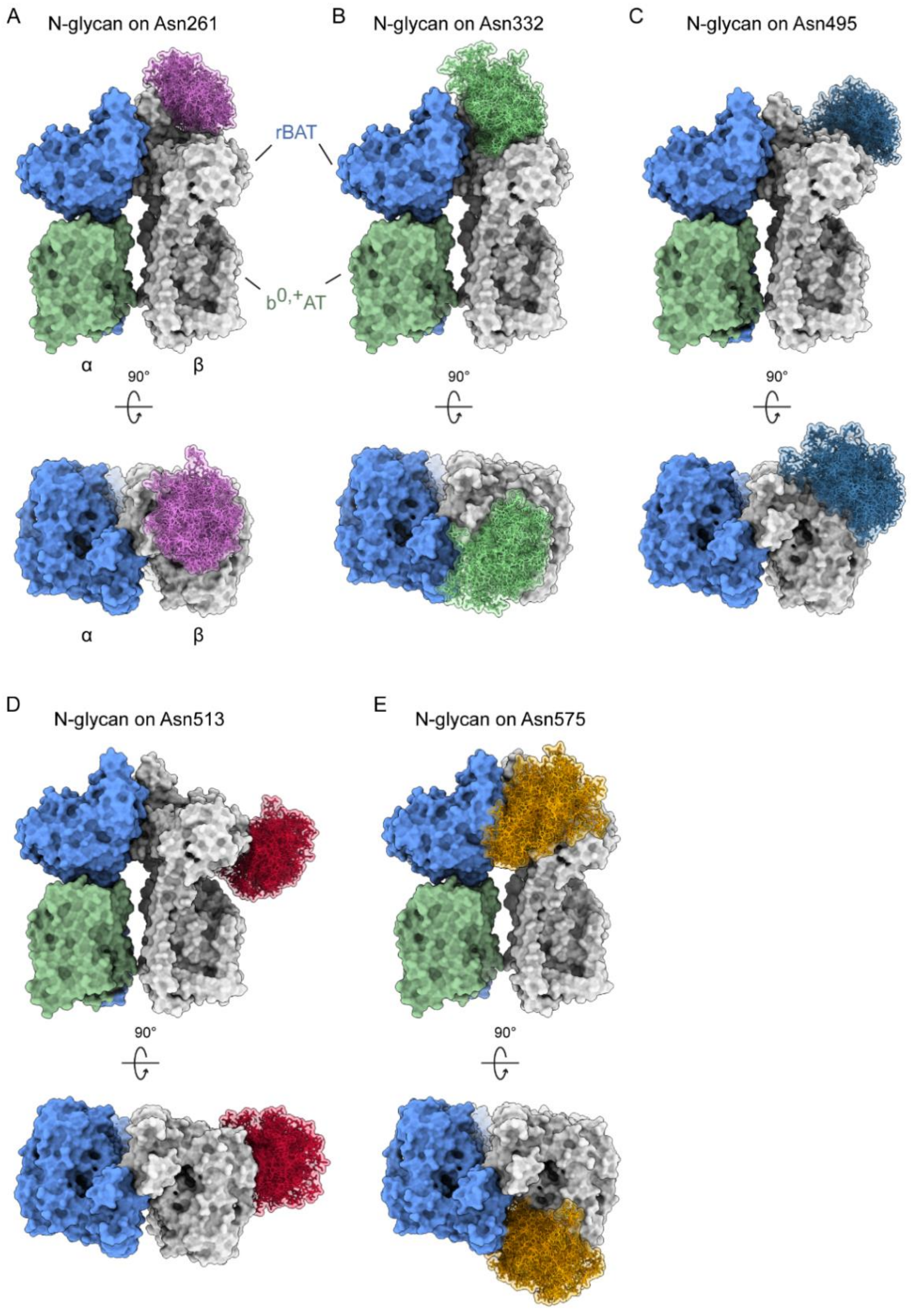

Supplementary Figure 18. Modelling N-glycans on the $b^{0,+}$AT-rBAT super-dimer (PDB:6LI9). The simulated N-glycan conformers on Asn261, Asn322, Asn495, Asn513 and Asn575 (panel A to E

respectively) in rBAT (β subunit) are highlighted in pink, green, blue, red and yellow, respectively. In panel E, the interfacial N-glycan on Asn575 contacts extensively the neighouring rBAT (α subunit).

Supplementary Methods

Detergent screening for native MS analysis. Recombinant LAT1-4F2hc assemblies (heterodimer and supr-dimer) from HEK293F cells were buffer-exchanged into 1M ammonium acetate buffer with glyco diosgenin (GDN), lauryl maltose neopentyl glycol (LMNG)/cholesteryl hemisuccinate (CHS), n-dodecyl-β-D-maltoside (DDM) and octyl glucose neopentyl glycol (OGNG) using Zeba spin desalting columns (7K MWCO, Thermo Fisher Scientific). The desalted protein complexes were then analyzed using a Q-Exactive UHMR mass spectrometer (Thermo Fisher Scientific).

Exoglycosidase treatment. Recombinant LAT1-4F2hc assemblies (100 µg) were then incubated with 20 units of α2-3,6,8,9 neuraminidase A (New England Biolabs) at 4 ºC overnight. The desialylated LAT1-4F2hc assemblies were then buffer-exchanged into 1 M ammonium acetate, pH 7.0 with 2 mM OGNG for native MS analysis.

Palmitoylation analysis. The LAT1-R183L mutant was incubated with 500 mM hydroxylamide in 1 M ammonium acetate, 36 µM GDN for 30 min. The sample was then buffer-exchanged into 1 M ammonium acetate with 2 mm OGNG for native MS analysis. Palmitoylation site prediction was performed using GPS-Palm software [1].

Protein delipidation. LAT1-4F2hc assemblies (200 µg) were buffer-exchanged into 1M ammonium acetate (pH 7.0) with 20 mM OGNG using a 100 kD MWCO centrifugal filter (Amicon Ultra-0.5 ml, Millipore) and incubated for 4 h at room temperature with gentle mixing. Excess lipomicelles and detergents were removed by buffer-exchanging into 1 M ammonium acetate with 2 mM OGNG using the same filter. The protein concentration was determined using a Nanodrop spectrophotometer (Thermo Scientific) before MS analysis.

Proteomics analysis. LAT1-4F2hc assemblies were incubated with 10 mM dithiothreitol (DTT) in 1× LDS SDS-PAGE sample buffer (Pierce) at 56 ºC for 5 min to reduce the disulfide bond between LAT1 and 4F2hc. The protein sample was then separated by NuPAGE 4 to 12%, Bis-Tris gel (Invitrogen). The LAT1 and 4F2hc gel bands were sliced, reduced again with 10 mM DTT at 56 ºC for 5 min, alkylated with 20 mM iodoacetamide (IAA) at room temperature for 20 min in the dark, and incubated with trypsin at 37 ºC overnight for proteolytic digestion. Digested peptides were dried using a SpeedVac vacuum concentrator (Thermo Fisher Scientific) and reconstituted with 1% formic acid for LC-MS/MS analysis. The tryptic peptides were analyzed on a Dionex Ultimate 3000 UHPLC coupled to an LTQ Orbitrap XL mass spectrometer (Thermo Fisher Scientific). Peptides were firstly loaded onto a 75 µm×2 cm pre-column and separated on a 75 µm×15 cm Pepmap C18 analytical column (Thermo Fisher Scientific) with a binary buffer system. Buffer A was 0.1% formic acid (FA) in 100% H_2O and buffer

B was 0.1% FA in 80% acetonitrile with 20% H_2O. The LTQ mass spectrometer was operated in data-dependant acquisition mode with one full MS scan (335 to 2000 m/z at a resolution of 60000) followed by MS/MS scans with the collision-induced dissociation (CID) normalized energy of 35%. The glycopeptide identification was performed manually using Xcalibur (version 4.1). The phosphopeptide identification was performed using Maxquant v2.1.0.

Lipidomics analysis. The LAT1-4F2hc complex (20 µg) was buffer-exchanged into 1M ammonium acetate, pH 7.0 with 2 mM OGNG and incubated with 1 µg trypsin overnight at 37 °C. One biological replicate was performed. The digested peptide/lipid mixture was dried using a SpeedVac vacuum concentrator (Thermo Fisher Scientific) and reconstituted with 70% mobile phase A (acetonitrile/H_2O: 60/40, 10 mM ammonium formate and 0.1% formic acid) and 30% mobile phase B (isopropanol/acetonitrile: 90/10, 10 mM ammonium formate and 0.1% formic acid) for the following LC-MS/MS analysis. Lipids were directly loaded onto a C18 column (Acclaim PepMap 100, C18, 75 µm × 15 cm, Thermo Scientific) by a Dionex UltiMate 3000 RSLC Nano system coupled to a LTQ Orbitrap mass spectrometer (Thermo Scientific). The lipids were separated with a gradient from 30% to 99 % mobile phase B. For data-dependent acquisition, full MS scans were acquired on the Orbitrap (m/z 400-2000) with a resolution of 60000. Collision-induced dissociation (CID) fragmentation in the ion trap was performed for the five most intense ions at an automatic gain control target of 30,000 and a normalized collision energy of 38%. Raw data were processed with MZmine v2.53 for phospholipid identification and quantification [2]. Lipid quantification for delipidated LAT1-4F2hc was performed manually using Xcalibur 4.4. The extracted ion chromatogram (XIC) of each lipid was processed with 20 ppm mass tolerance and a 7-point Gaussian smoothing. The area under the curve (AUC) was integrated for lipid quantification.

***In silico* simulation.** The sequences of LAT1 and 4F2hc were submitted to AlphaFold Colab (v1.5.2) [3] for LAT1 homodimer and LAT1-4F2hc super-dimer structure prediction using AlphaFold2-multimer [4,5]. For simulation of glycan conformers, the non-glycosylated protein structures were retrieved from RCSB PDB database and prepared using CHARM-GUI [6]. The simulation of glycan conformers on LAT1-4F2hc was performed using GlycoSHIELD (v 0.1) [7]. ChimeraX 1.2.5 was used for protein structure visualization [8].

Data analysis. Simulation and visualization of the pseudo-spectrum of the LAT1-4F2hc super-dimer was performed using Jupyter Notebook with Python 3 and Seaborn library [9]. Statistical analysis was performed using Prism 8.0 (GraphPad Software Inc., San Diego, CA).

Cell culture. HeLa (CCL-2, ATCC) and A431 (CRL-1555, ATCC) cells were cultured in Dulbecco's Modified Eagle's Medium (DMEM) (Gibco) supplemented with 10 % fetal bovine serum (FBS) and grown at 37 °C under 5 % CO_2. HepG2 (HB-8065, ATCC) cells were cultured in Minimum Essential Medium (MEM) supplemented under 10 % FBS and grown at 37 °C with 5 % CO_2. The lysosome purification was performed using a lysosome enrichment kit from Thermo Scientific (catalog number: 89839).

In vivo cross-linking. HeLa cells were harvested and washed three times with ice-cold DPBS (pH 8.0, Gibco, Thermo Fisher Scientific). Cells were then suspended in PBS (pH 8.0) at 2.5×10^7 cells/mL and mixed with freshly prepared BS^3 reagent (Thermo Scientific) at a final concentration of 1 mM at room temperature. After 45 min incubation, the reaction was quenched by adding 500 mM Tris buffer (pH 7.4) to a final concentration of 20 mM. After 15 min incubation, the cells were lysed for Western blotting or affinity-purfication experiments.

Preparing the membrane fraction for affinity-purification and Western blotting. A431, HeLa and HepG2 cells were cultured in DMEM and MEM medium (Gibco, Thermo Fisher Scientific) at 37 °C under 5% CO_2, until the cell confluency reached 70%. The cells were then harvested and washed three times with ice-cold DPBS (Gibco, Thermo Fisher Scientific). The cells (packed cell volume of 0.2 mL) were then suspended in 0.6 mL hypotonic buffer (10 mM HEPES, 1 mM EGTA, 25 mM KCl, pH 7.8) and incubated at 4 °C for 20 min. After centrifugation at 600 g for 10 min, the cells were suspended in two times the volume of the packed cell in isotonic buffer (10 mM HEPES, 1 mM EGTA, 25 mM KCl and 250 mM sucrose, pH 7.8) with EDTA-free protease inhibitor cocktail (Roche). Then, the cells were homogenized and centrifuged at 1000 g for 10 min at 4 °C to remove the nuclei and unbroken cells. The supernatant was collected and centrifuged again at 12000 g for 15 min at 4 °C to remove the mitochondrial fraction. The supernatant was diluted 2-fold with PBS and further centrifuged at 150000 g to pellet the membranes for the following affinity-purification and Western blotting experiments.

Affinity-purification of cross-linked endogenous LAT1-4F2hc complexes. Membranes from HeLa cells were incubated with TBS buffer (50 mM Tris, 150 mM NaCl, pH 7.4) with 1% DDM at 4 °C for 2 hours then centrifuged at 12000 g for 10 min to remove unsolubilized particles. The supernatant (solubilized membrane proteins) was collected and diluted with TBS buffer to a final concentration of 0.2 % DDM. Solubilized membrane proteins were then incubated with 50 µL Anti-LAT1 antidbody (#5347, Cell Signaling Technology) at 4 °C overnight; then incubated with 50 µL Protein A agarose beads (#9863, Cell Signaling Technology) at 4 °C for 2 hours. After washing three times with TBS buffer containing 0.1% DDM, the beads were incubated with 20 µL 2 × NuPAGE LDS sample buffer (Pierce) at 95 °C for 5 min and loaded to SDS-PAGE gel for separation.

Western blotting of endogenous LAT1-4F2hc assemblies. The membranes of A431, HeLa and HepG2 cells were incubated with 1× NuPAGE LDS sample buffer (Pierce) for 30 min at room temperature and centrifuged at 12000 g for 5 min to remove undissolved particles. To reduce the disulfide bond in the LAT1-4F2hc complex, the samples in LDS sample buffer were further incubated with 10 mM DTT at 56 °C for 10 min. Membrane proteins were then separated by NuPAGE 4 to 12%, Bis-Tris gel (Invitrogen) and transferred to PVDF membrane (Invitrogen). The PVDF membrane was then blocked with 5 % BSA (Fraction V, Roche) in TBST buffer (50 mM Tris, 150 mM NaCl and 0.1% Tween-20) and incubated with primary antibodies diluted 1000 fold with TBST supplemented with 0.1% BSA overnight at 4 °C. The following antibodies Anti-LAT1 (#5347), Anti-4F2hc antibody

(#13180) and Anti-LAMP1 (#9091) were obtained from Cell Signaling Technology and used to probe for LAT1, 4F2hc and LAMP1, respectively. After three washes, the membranes were incubated with 2000 fold diluted HRP-linked anti-rabbit IgG antibody (#7074, Cell Signaling Technology) for 1 h at room temperature. The membranes were developed using enhanced chemiluminescence (ECL) Western blotting substrate (Pierce) and scanned with Bio-Rad ChemiDoc XRS+ imaging system.

Supplementary References

1. Ning, W. *et al.* GPS-Palm: A deep learning-based graphic presentation system for the prediction of S-palmitoylation sites in proteins. *Brief. Bioinform.* **22**, 1836–1847 (2021).
2. Pluskal, T., Castillo, S., Villar-Briones, A. & Orešič, M. MZmine 2: Modular framework for processing, visualizing, and analyzing mass spectrometry-based molecular profile data. *BMC Bioinformatics* **11**, 395 (2010).
3. Mirdita, M. *et al.* ColabFold: making protein folding accessible to all. *Nat. Methods* **19**, 679–682 (2022).
4. Evans, R. *et al.* Protein complex prediction with AlphaFold-Multimer. *bioRxiv* 2021.10.04.463034 (2022) doi:10.1101/2021.10.04.463034.
5. Jumper, J. *et al.* Highly accurate protein structure prediction with AlphaFold. *Nature* **596**, 583–589 (2021).
6. Jo, S., Kim, T., Iyer, V. G. & Im, W. CHARMM-GUI: A web-based graphical user interface for CHARMM. *J. Comput. Chem.* **29**, 1859–1865 (2008).
7. Gecht, M. *et al.* GlycoSHIELD: a versatile pipeline to assess glycan impact on protein structures. *Prepr. bioRxiv* 2021.08.04.455134 (2021) doi:10.1101/2021.08.04.455134.
8. Pettersen, E. F. *et al.* UCSF Chimera--a visualization system for exploratory research and analysis. *J. Comput. Chem.* **25**, 1605–12 (2004).
9. Waskom, M. Seaborn: Statistical Data Visualization. *J. Open Source Softw.* **6**, 3021 (2021).
10. Zhang, C. *et al.* SLC3A2 N-glycosylation and Golgi remodeling regulate SLC7A amino acid exchangers and stress mitigation. *J. Biol. Chem.* 105416 (2023) doi:10.1016/j.jbc.2023.105416.
11. Stetsenko, A., Guskov, A. & Version, D. An Overview of the Top Ten Detergents Used for Membrane Protein Crystallization. *Crystals* **7**, 197 (2017).
12. Roth, A. F., Wan, J., Green, W. N., Yates, J. R. & Davis, N. G. Proteomic identification of palmitoylated proteins. *Methods* **40**, 135–142 (2006).
13. Krissinel, E. & Henrick, K. Inference of Macromolecular Assemblies from Crystalline State. *J.*

Mol. Biol. **372**, 774–797 (2007).

14. Gupta, K. *et al.* The role of interfacial lipids in stabilizing membrane protein oligomers. *Nature* **541**, 421–424 (2017).

15. Janson, G. & Paiardini, A. PyMod 3: a complete suite for structural bioinformatics in PyMOL. *Bioinformatics* **37**, 1471–1472 (2021).

16. Nusinow, D. P. *et al.* Quantitative Proteomics of the Cancer Cell Line Encyclopedia. *Cell* **180**, 387-402.e16 (2020).
