# Supplementary material for: The complete assembly of human LAT1-4F2hc complex provides insights into its regulation, function and localisation
Source: Nat Commun. 2024 May 2;15:3711. doi: 10.1038/s41467-024-47948-4 (PMC11065870; doi:10.1038/s41467-024-47948-4)
Supplement: Supplementary file 2 — Reporting Summary [file 41467_2024_47948_MOESM2_ESM.pdf]

nature portfolio

Corresponding author(s): Di Wu & Carol V. Robinson

Last updated by author(s): Mar 5, 2024

Reporting Summary

Nature Portfolio wishes to improve the reproducibility of the work that we publish. This form provides structure for consistency and transparency in reporting. For further information on Nature Portfolio policies, see our Editorial Policies and the Editorial Policy Checklist.

Statistics

For all statistical analyses, confirm that the following items are present in the figure legend, table legend, main text, or Methods section.

n/a	Confirmed	
☐	☒	The exact sample size (*n*) for each experimental group/condition, given as a discrete number and unit of measurement
☐	☒	A statement on whether measurements were taken from distinct samples or whether the same sample was measured repeatedly
☐	☒	The statistical test(s) used AND whether they are one- or two-sided<br>*Only common tests should be described solely by name; describe more complex techniques in the Methods section.*
☒	☐	A description of all covariates tested
☒	☐	A description of any assumptions or corrections, such as tests of normality and adjustment for multiple comparisons
☐	☒	A full description of the statistical parameters including central tendency (e.g. means) or other basic estimates (e.g. regression coefficient) AND variation (e.g. standard deviation) or associated estimates of uncertainty (e.g. confidence intervals)
☐	☒	For null hypothesis testing, the test statistic (e.g. *F*, *t*, *r*) with confidence intervals, effect sizes, degrees of freedom and *P* value noted<br>*Give P values as exact values whenever suitable.*
☒	☐	For Bayesian analysis, information on the choice of priors and Markov chain Monte Carlo settings
☒	☐	For hierarchical and complex designs, identification of the appropriate level for tests and full reporting of outcomes
☐	☒	Estimates of effect sizes (e.g. Cohen's *d*, Pearson's *r*), indicating how they were calculated

Our web collection on statistics for biologists contains articles on many of the points above.

Software and code

Policy information about availability of computer code

Data collection	Q Exactive UHMR (Thermo Fisher); LTQ-Orbitrap XL (Thermo Fisher); Refeyn TwoMP mass photometer (Refeyn Ltd); Bio-Rad ChemiDoc XRS+ imaging system. (Bio-Rad)
Data analysis	UniDec v5.1.1 (https://github.com/michaelmarty/UniDec/), iFAMs (https://github.com/prellgroup/iFAMS), Xcalibur v4.1.50 (Thermo Fisher), ChimeraX 1.2.5, AlphaFold Colab v1.5.2, CHARM-GUI (https://www.charmm-gui.org/), GlycoSHIELD v0.1 (https://github.com/GlycoSHIELD-MD/GlycoSHIELD-MD), GPS-Palm (https://gpspalm.biocuckoo.cn/), Mzmine v2.53 (https://github.com/mzmine/mzmine2/), Maxquant v2.1.0, GraphPad Prism 8.0, AcquireMP v2.3, DiscoverMP v2.3, Jupyter Notebook with Python 3

For manuscripts utilizing custom algorithms or software that are central to the research but not yet described in published literature, software must be made available to editors and reviewers. We strongly encourage code deposition in a community repository (e.g. GitHub). See the Nature Portfolio guidelines for submitting code & software for further information.

Data

Policy information about availability of data

All manuscripts must include a data availability statement. This statement should provide the following information, where applicable:

- Accession codes, unique identifiers, or web links for publicly available datasets
- A description of any restrictions on data availability
- For clinical datasets or third party data, please ensure that the statement adheres to our policy

The raw native MS data that generated in this study have been deposited in the Figshare database doi:10.6084/m9.figshare.24139461. The proteomics and

lipidomics raw data that generated in this study have been deposited in the MassIVE database under the accession code MSV000094478 [ftp://massive.ucsd.edu/v07/MSV000094478/]. The atomic coordinates for structural illustration and simulation are available in the PDB database under accession code 6IRT [http://doi.org/10.2210/pdb6irt/pdb] (LAT1-4F2hc) and 6LI9 [http://doi.org/10.2210/pdb6li9/pdb] (b0,+AT-rBAT). Source data are provided with this paper.

Research involving human participants, their data, or biological material

Policy information about studies with human participants or human data. See also policy information about sex, gender (identity/presentation), and sexual orientation and race, ethnicity and racism.

Reporting on sex and gender	N/A
Reporting on race, ethnicity, or other socially relevant groupings	N/A
Population characteristics	N/A
Recruitment	N/A
Ethics oversight	N/A

Note that full information on the approval of the study protocol must also be provided in the manuscript.

Field-specific reporting

Please select the one below that is the best fit for your research. If you are not sure, read the appropriate sections before making your selection.

☒ Life sciences ☐ Behavioural & social sciences ☐ Ecological, evolutionary & environmental sciences

For a reference copy of the document with all sections, see nature.com/documents/nr-reporting-summary-flat.pdf

Life sciences study design

All studies must disclose on these points even when the disclosure is negative.

Sample size	No sample size determination was needed. All proteins were selected based on underlining biology and availability.
Data exclusions	No data exclusion was needed
Replication	For mass spectrometry and mass photometry quantification, each measurement was replicated 3 times. All attempts at replications were successful and a representative spectrum of each sample was displayed in all cases. For lipidomics and proteomics identification, all data have been successfully repeated with at least two batches of samples. Each Western blotting experiment was performed once.
Randomization	This is not relevant to this study, because no grouped samples was involved.
Blinding	This is not relevant to this study, because no grouped samples was involved.

Reporting for specific materials, systems and methods

We require information from authors about some types of materials, experimental systems and methods used in many studies. Here, indicate whether each material, system or method listed is relevant to your study. If you are not sure if a list item applies to your research, read the appropriate section before selecting a response.

Materials & experimental systems

n/a	Involved in the study	
☐	☒	Antibodies
☐	☒	Eukaryotic cell lines
☒	☐	Palaeontology and archaeology
☒	☐	Animals and other organisms
☒	☐	Clinical data
☒	☐	Dual use research of concern
☒	☐	Plants

Methods

n/a	Involved in the study	
☒	☐	ChIP-seq
☒	☐	Flow cytometry
☒	☐	MRI-based neuroimaging

Antibodies

Antibodies used	LAT1 antibody (Cell Signaling Technology, #5347); 4F2hc/SLC3A2 (D6O3P) antibody (Cell Signaling Technology, #13180); LAMP1

	(D2D11) XP Antibody (Cell Signaling Technology, #9091); Anti-rabbit IgG, HRP-linked Antibody (Cell Signaling Technology, #7074); anti-FLAG M2 affinity resin (Sigma, #A2220)
Validation	https://www.cellsignal.com/products/primary-antibodies/lat1-antibody/5347<br>https://www.cellsignal.com/products/primary-antibodies/4f2hc-cd98-d6o3p-rabbit-mab/13180<br>https://www.cellsignal.com/products/primary-antibodies/lamp1-d2d11-xp-rabbit-mab/9091<br>https://www.cellsignal.com/products/secondary-antibodies/anti-rabbit-igg-hrp-linked-antibody/7074<br>https://www.sigmaaldrich.com/GB/en/product/sigma/a2220

Eukaryotic cell lines

Policy information about cell lines and Sex and Gender in Research

Cell line source(s)	HeLa (ATCC), HepG2 (ATCC), A431 (ATCC), HEK293F (Invitrogen)
Authentication	No further authentication was performed for commercially availabe cell lines.
Mycoplasma contamination	Not tested for mycoplasma contamination.
Commonly misidentified lines (See ICLAC register)	No commonly misidentified cell lines were used.

Plants

Seed stocks	N/A
Novel plant genotypes	N/A
Authentication	N/A
